# Exploring competencies of military nurses in general hospitals in China: a qualitative content analysis

**DOI:** 10.1186/s12912-021-00673-5

**Published:** 2021-08-23

**Authors:** Huijuan Ma, Li Lin, Suofei Zhang, Lei Lei, Jinyu Huang, Fang Lu, Yu Luo

**Affiliations:** 1 School of Nursing, Third Military Medical University/Army Medical University, Chongqing, P.R. China; 2 Institute of Military Preventive Medicine, Third Military Medical University/Army Medical University, Chongqing, P.R. China

**Keywords:** Competency, Military nurse, Qualitative content analysis

## Abstract

**Background:**

Military nurses should possess the competency to provide quality care in both clinical and military nursing contexts. This study aimed to identify the competencies of military nurses in general hospitals.

**Methods:**

A qualitative study was carried out using a qualitative content analysis. We purposefully sampled and interviewed 21 nurses in general hospitals in China.

**Results:**

The data analysis revealed 40 competencies, which were categorised into four main categories according to the Onion Model. These categories were motive (mission commitment), traits (perseverance, flexibility, etc.), self-identity of dual roles (obedience, empathy, etc.), as well as knowledge, skills and abilities (clinical and military nursing knowledge and skills, basic nursing ability, professional development ability, leadership and management ability).

**Conclusions:**

Existing knowledge of competencies of military nurses in general hospitals is limited. A detailed exploration of this topic can provide guidance for recruitment, competency assessment, and competency building.

## Background

Competency is defined as a personal trait or set of habits that leads to more effective or superior job performance, and it has five major components: knowledge, skills, self-concepts and values, traits, and motives [1, 2]. Motives are initiators that prompt people to do their work, and traits refer to personal qualities [3]. Self-concepts and values refer to self-identity, which includes a person’s attitudes and self-image, while knowledge and skills are fundamental requirements for a person to perform a certain task or job [3].

The responsibilities of military nurses are slightly different from those of their civilian counterparts as the former are indispensable during wars and United Nations (UN) peacekeeping missions. Frequently required to respond to natural disasters or epidemics to save lives [4], they play dual roles as both military officers and nurses [5]. The scope of practice for military nurses working in general hospitals includes not only routine nursing tasks but also military missions. The military nursing context is characterised by trauma-centred care to patients of all ages, as well as harsh conditions, which include the potential for physical and psychological harm [6–9]. The instability of this healthcare environment and demanding operational requirements increase military nurses’ burden [5]. Even when working in general hospitals, military nurses require the competencies to be able to thrive in this environment.

Owing to rapid population ageing and changes in disease presentations, while the healthcare environment has been greatly altered, the requirement for nurses remains the same [10]. Nurses are expected to possess the abilities to provide comprehensive, quality care. To accomplish this, it is important for nurses to develop nursing competencies, which are the core abilities that are required for fulfilling their roles [11]. In other words, nursing competencies are the necessary knowledge, skills, and attitudes nurses must possess to perform their duties in a safe and ethical manner. The American Association of Colleges of Nursing outlines a set of nursing competencies and highlights such areas as ‘patient-centered care, interprofessional teams, patient safety, informatics, critical thinking, cultural sensitivity, and professionalism’ [12]. Meanwhile, military nursing competencies encompass clinical nursing competency, operational nursing competency, soldier/survival skills, personnel/physical/psychosocial stress, leadership and administrative support, and group integration and identification [13].

Models have been developed to apply these competencies to workforce performance. One example is the multilayered Onion Model. The outer layer of the Onion Model can easily be seen and cultivated, while the inner layer is difficult to evaluate, making training in this aspect also difficult [2]. Knowledge, skills, self-concepts, values, traits, and motives are divided into three layers, moving from the outer layer towards the core. More specifically, the outer layer contains knowledge and skills, the middle layer of self-identity includes self-concepts and values, and the core of the Onion Model encompasses traits and motives. Based on the evidence that competencies can be used for translating strategy into job-related performance and individual behaviours, the development and application of a competency model enables the cultivation of a more effective and productive workforce [14].

While the literature on general nursing competencies is vast, research focusing on the competencies of military nurses is limited. The Onion Model is an enriched competency model with different interrelated layers, which can provide a comprehensive perspective of exploring competencies and designing multi-level training. In order to gain a better understanding of military nurses’ competencies, we conducted a qualitative study on the theoretical basis of the Onion Model, which can provide enriched theoretical guidance for competency-based nursing education and competency building.

## Methods

### Design

A qualitative study was carried out using a qualitative content analysis and conducted from April to June 2020 [15]. This design provides a contextual description and interpretation of social phenomena [16]—in this case the competencies of military nurses in general hospitals—and facilitates an understanding of related voices, views, and thoughts. The reporting of the study was based on the consolidated criteria for reporting qualitative research (COREQ) checklist [17].

### Participants

Based on the requirements of qualitative studies, a small convenience sample was solicited [18]. Military nurses from general hospitals in China with experience of participating in military missions such as disaster and public health emergency rescue were included. Nurses from 10 military general hospitals were contacted to determine their willingness to participate in this study. Written informed consent was obtained from each participant. In total, we interviewed 21 nurses until data saturation was reached.

### Data Collection

After obtaining institutional permission, telephone or WeChat (a popular Chinese social media app) conversations were conducted between the research team and military nurses, during which the nurses were introduced to the study objectives and asked if they were willing to participate. Willing nurses were then given an explanation of the purpose, significance, and confidentiality of the study and informed that they could withdraw at any time. Meanwhile, a formal interview was scheduled at the convenience of the participant. The interview outline was based on the Onion Model and behavioural event interview framework [1, 2]. The following questions were asked, followed by probing questions: (1) background questions including age, gender, qualification, job title, years of working as a military nurse, and name of institution; (2) experience of being deployed in military missions, and the most impressive events—both successful and unsuccessful—during each deployment; and (3) competencies of military nurses. The interviews were conducted in Mandarin, digitally recorded and uploaded to an online transcription service. Each interview lasted approximately one hour, and notes were taken in each interview.

### Data Analysis

Data were analysed using an inductive approach combined with a deductive approach, and Graneheim and Lundman’s qualitative content analysis technique was utilised [15]. Upon completion of each interview, the recordings were transcribed. The research team read the text several times for a general understanding and then outlined the meaning of units and phrases that were significant to the topic. Every meaning unit was condensed and labelled with a code and subsequently classified into categories and subcategories based on similarities. A deductive approach was used to relate the categories and subcategories to the three layers of the Onion Model. After identifying categories and subcategories, these categories, subcategories and corresponding quotes were independently translated into English by two bilingual researchers. Any disagreements were discussed and resolved with another bilingual researcher.

Data trustworthiness should be achieved among qualitative research findings, and trustworthiness was ensured through four aspects: credibility, dependability, confirmability, and transferability [15]. To achieve credibility, agreement was sought among research team when recruiting participants, selecting suitable meaning units, and classifying categories. Additionally, our research team is knowledgeable and experienced with regard to qualitative research and data analysis. To ensure dependability, confirmability and transferability, a transparent methodological process was followed, and a thorough description of quotes was integrated with the findings.

### Ethical considerations

This study protocol was submitted to the Medical Ethics Committee of Army Medical University in Chongqing, China. The research was approved by the Medical Ethics Committee and determined to be exempt. Prior to data collection, participants received an information letter about the study and a consent form. This study was carried out under the willingness of participants who signed a consent form before participating in this study.

## Results

Table 1 depicts the participants’ demographic characteristics. The final sample consisted of one male and 20 female military nurses from 10 military hospitals in China. The average age was 35 ± 6.32 years (range: 24–46) and the average years of working was 14 ± 7.78 years (range: 2–30). Five nurses held master’s or doctoral degrees, and 16 others held a bachelor’s degree. The military missions they had participated in included disaster rescue, Ebola virus in Africa, UN peacekeeping operations, public emergencies, and drills.

**Table. 1:** Demographic characteristics of participants

Variable	*n*	%
Gender		
Female	20	95.2
Male	1	4.8
Age		
Less than 30 years old	7	33.3
31 to 40 years old	10	47.6
More than 40 years old	4	19.0
Year of working as nurse		
Less than 10 years	6	28.6
11 to 20 years	10	47.6
More than 20 years	5	23.8
Current Level of Education		
Bachelors	16	76.2
Masters	4	19.0
Doctoral	1	4.8
Title		
Nurse practitioner	5	23.8
Nurse in charge	11	52.4
Associate senior nurse	5	23.8
Position		
Nurse	7	33.3
Head Nurse	12	57.2
Chief of nursing department	2	9.5
Participation in military mission		
Disaster rescue	5	16.7
United Nations peace-keeping operation	5	16.7
Public emergency	6	20.0
Drill	14	46.7

**Fig. 1:**
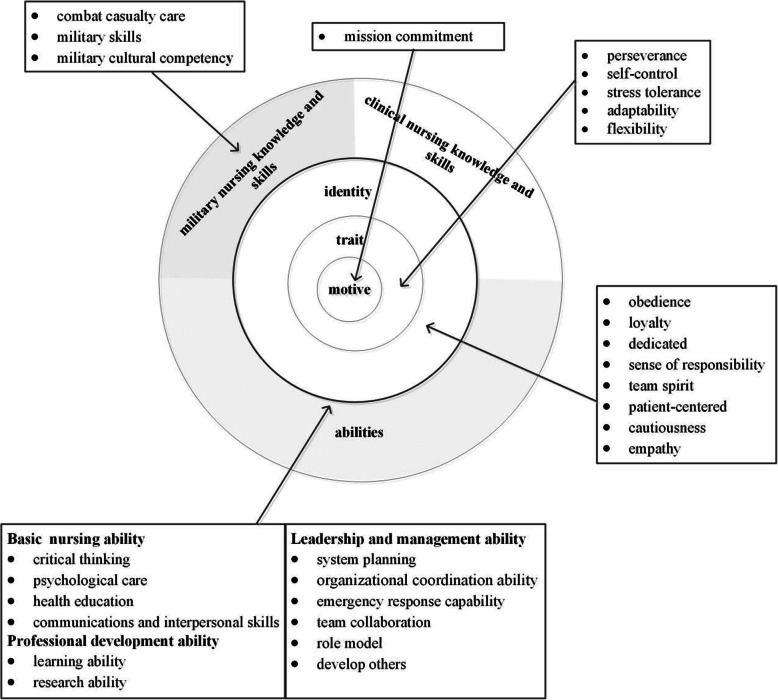
The onion competency model of military nurses in general hospitals

From the qualitative data analysis, 40 competencies were identified and divided into four main categories including motive, traits, self-identity, and knowledge, skills, and abilities, as shown in the onion competency model of military nurses in general hospitals (Fig. 1).

### Motive

Motive is the core of the onion competency model of military nurses in general hospitals. Mission commitment, the root of personal inspiration, was identified as the primary motive among participants in this study. Participants expressed that their identity as military nurses motivated them to actively commit to military missions.

*When my hospital assigned me, I was a little hesitant because my child was breastfeeding. However, I still participated considering I was needed in this mission. (P07)*

*I voluntarily applied to participate in the UN peacekeeping mission. I wanted to participate from my heart, and I want to have more such experiences to fulfil my military career. (P04)*

### Traits

Traits formed the second layer from the core of the onion competency model. Perseverance, self-control, stress tolerance, adaptability, and flexibility, which are the character strengths of military nurses, were the important traits identified in this study.

*The temperature there was 40 degrees. We walked constantly for four hours and treated lots of soldiers in this weather. We finally completed this mission with perseverance. (P10)*

*I think stress tolerance is important, especially when you work in a hostile environment such as the Gobi desert for months. Some people did not adapt well when they were far away from family and original work, and they showed symptoms such as anxiety and depression. (P03)*

*If you are going to the battlefield to save lives, you need to have flexibility. This is because the nursing context you encounter would be more variable than the environment of work in hospital. (P20)*

### Self-identity

The middle layer is the self-identity of being a military nurse. Military nurses play dual roles as both military officers and nurses, indicating that they should possess values and self-concepts relevant to both identities.

As military officers, the participants in this study emphasised the prioritisation of the values of the military and the importance of obedience, loyalty, dedication, a sense of responsibility, and team spirit.

*As military nurses, we should obey orders, and we should also be brave when confronting difficulties. I think a sense of responsibility is fundamental to being a military nurse. (P02)*

*I would like to persist to the end of this mission, even if I feel weak after a large amount of physical exertion, because this is a team, and what I do is for the honour of the team. (P09)*

Military nurses should also show professional values and attitudes towards patients. Patient-centredness, cautiousness, and empathy were identified in this study.

*When I entered the room of one Ebola patient, I found he had a fever and was in pain. When I gave him the oral medicine, he refused to take it because of his bad appetite. Because these medicines are necessary, I talked to him patiently, and when he was willing to eat, I helped him swallow the medicines one by one. (P05)*

*I treated work seriously, and I did everything I could remind myself to do. I did not relax and lower my standards even when no one was supervising my work. (P18)*

### Knowledge, skills, and abilities

The outer layer of the Onion Model is composed of knowledge and skills, along with the supporting abilities. Military nurses working in general hospitals in China might face two kinds of nursing contexts; one is the daily routine care that is similar to the job responsibilities of a civilian nurse, and the other is the military nursing context during healthcare missions. In order to distinguish between different nursing scenarios, the outer layer is separated into the knowledge, skills, and abilities related to clinical and military nursing.

*Because each one works in different departments, such as the outpatient department, surgical department, and operating room, the professional quality of each department is very important during daily routine work. (P15)*

In the military context, combat casualty care, military skills, and military cultural competency were highlighted. Combat casualty care is focused on trauma care for a wide range of patients from children to older adults. Military skills encompass chemical, biological, and radiological protection, security awareness, and soldier skills. Military cultural competency enables military nurses to provide quality nursing care to people from diverse backgrounds.

*Combat casualty care is different to the daily routine care in hospital, for example, when caring for a heart stroke patient in the battlefield and facing multiple other patients, you might make different decisions from what you make in peacetime. (P17)*

*When I failed to put an IV bag for a paediatric patient and one of my colleagues from the department of paediatrics succeeded, I felt depressed. (P12)*

The importance of abilities related to basic nursing, professional development, and leadership and management was also identified.

Basic nursing ability is composed of critical thinking, communication and interpersonal skills, and psychological care, together with health education. Critical thinking is an essential process for a safe, efficient, and skilful nursing intervention, while communication and interpersonal skills are fundamental for nursing work. Psychological care is essential, especially when facing patients with posttraumatic stress disorder, and health education helps patients expand their knowledge of relevant diseases.

*When the patient with stress combat syndrome came, I did not know how to cope with the condition, and I just stayed there with this patient. I was not satisfied with my way of coping, and I realised I lacked the knowledge and skills of psychological nursing. (P01)*

*When deployed to special environments like plateaus, you should know the common diseases in this environment. You not only have to prevent yourself from being ill but also instruct soldiers on being healthy; thus, you should have the ability to impart health education. (P14)*

Professional development ability includes learning and research skills, which enable and promote stable professional development.

*The reason I took part in military nursing was that I wanted to find my weakness through the context. Then I could learn from this lesson and go on to professional learning, and this was a good process for my personal growth and professional development. (P03)*

*We lack research ability because we can learn a lot from the tasks. Thus, we can learn from our experiences and do better in the next task. (P13)*

Leadership and management ability encompasses system planning, organisational coordination, team collaboration, emergency response, role modelling, and encouraging others’ development.

*When you lead the team, system planning is essential, and you have to forecast what might happen and make relevant protocols. (P05)*

*I think organisational and management ability is important, and when something happens, nurses should know how to deal with the situation, including rational coordination and arrangement of other people, instead of acting with confusion and not knowing their position. (P11)*

*The senior nurses really took good care of the junior nurses. A 54-year-old nurse applied to go to the frontline of the flood and asked young nurses to step back. I should learn from her to be a role model. (P12)*

## Discussion

Nursing in military hospitals is demanding, largely because of role duality; nurses are required to perform daily routine work as well as participate in military missions. While their daily routines are similar to those of civilian nurses, participating in military healthcare missions demands more competencies, especially in combat casualty care. Military nurses not only have to maintain their clinical competencies but also master soldier skills, and are expected to advance in leadership throughout the career ladder [19]. The competencies identified in this study are consistent with previous findings concerning nursing competencies [20]; further, the results present the competencies unique to military nurses. At the meantime, the Onion Model provided a good theoretical basis for this study because of the division of different elements into three layers, facilitating a clear understanding of competencies, which can contribute to competency-based nursing education.

Compared to civilian nurses, the duality of roles of military nurses indicates differences in their work environment, work values, missions, and education, which can influence the development of self-identity [21]. For instance, the motive of the interviewed military nurses in this study was mission commitment from their self-consciousness of their responsibilities, while the motive of civilian nurses could be respect from the healthcare team and life security [22]. The professional identity and values of nurses are the foundation for nursing practice, including empathy and responsibility which could be cultivated by nursing curriculum [23–25]. Meanwhile, the self-identity of military nurses is twofold; they relate to the above outlined nursing values as well as the values of being a military officer, which include obedience, loyalty, and dedication. Through military training, nurses are separated from civilian life and develop a strong identification with the military culture, which is characterised by loyalty, integrity, courage, determination, and a commitment to duty [26, 27]. Thus, they develop into brave and loyal military officers with strong faith and team spirit.

Rich competencies, which focused on military nursing and leadership skills, formed the outer layer in this study. These are in line with a framework of military nurse managers’ competencies, which encompasses clinical expertise, leadership competencies, deployment competencies, and so on [28]. Although one-third of participants were under 30 years old and did not work as leaders in the nursing team, most participants mentioned management skills and leadership. All nurses have a substantial role in leadership, and they are encouraged to explore the concept of leadership in the constantly changing field of healthcare [29]. Moreover, leadership is a core competency for all military nurses, which can be developed gradually from the unit level to the organisational level and finally to the strategic level [30].

Military nursing knowledge and skills in this study included combat casualty care, military skills, and military cultural competency. Tactical combat casualty care is a compulsory course for military nursing undergraduate students; it provides training on care under fire, tactical field care, and tactical evacuation care [31]. Besides combat casualty care, providing healthcare in field hospitals requires a multitude of specialties, including critical care, surgical care, and paediatric care [32]. The interviewed nurses in this study had experience of caring for patients of all ages and who had diverse diseases such as contagious illness and trauma. One participant expressed that she felt depressed after failing to administer an intravenous infusion to a paediatric patient; other studies have also shown that military nurses find it challenging to care for paediatric patients [8]. This finding indicates that an excellent military nurse should have clinical expertise with comprehensive clinical capabilities [28]. Additionally, around 30 % of participants in this study had experience of UN peacekeeping operations and fighting the Ebola virus in Africa, and they emphasised the importance of military cultural competency. Military cultural competency is especially important when providing quality nursing care to people from diverse backgrounds. Therefore, it is important to assess the skills, attitudes, and knowledge related to military cultural competency [33].

There are limitations to this study. First, using the Onion Model as the theoretical framework for the interview outline and deductive approach to data analysis might have led to some biases. In order to minimise the potential bias, the data analysis combined the inductive and deductive approaches. Second, the findings are context- and time-dependent and therefore cannot be generalised to all military nurses.

## Conclusions

Military nurses play a substantial role in providing quality nursing care to meet the demands of patients and the requirements of the ever-changing healthcare system. Existing knowledge of the competencies of military nurses in general hospitals is limited, and qualitative studies of deployment experiences of military nurses reveal that they feel unprepared, necessitating appropriate prior training [7]. The present findings contribute to a richer knowledge of nursing competencies for personnel recruitment and competency assessment and building. Future studies are necessary to design scientific assessment tools for military nursing competencies and effective vocational courses for leadership and professional development of military nursing teams. Furthermore, research exploring how to prepare military nurses to cope with deployment, which will promote the development of a competent, resilient, and prepared nursing workforce, is required.

## Data Availability

The datasets used and/or analysed during the current study are available from the corresponding author on reasonable request.

## Abbreviations

COREQ: Consolidated criteria for reporting qualitative research
UN: United Nations

## References

[1] McClelland DC (1973). Testing for competence rather than for intelligence. Am Psychol.

[2] Spencer L, Spencer S (1993). Competence at work: model for superior performance.

[3] Tucker SA, Cofsky KM (1994). Competency-based pay on a banding platform: a compensation combination for driving performance and managing change. ACA Journal.

[4] Michaud J, Moss K, Licina D, Waldman R, Kamradt-Scott A, Bartee M, Lim M, Williamson J, Burkle F, Polyak CS, Thomson N, Heymann DL, Lillywhite L (2019). Militaries and global health: peace, conflict, and disaster response. The Lancet.

[5] Griffiths L, Jasper M (2008). Warrior nurse: duality and complementarity of role in the operational environment. J Adv Nurs.

[6] Butler FK (2017). Tactical combat casualty care: beginnings. Wilderness Environ Med.

[7] Conlon L, Wiechula R, Garlick A (2019). Hermeneutic phenomenological study of military nursing officers. Nurs Res.

[8] Goodman P, Edge B, Agazio J, Prue-Owens K (2013). Military nursing care of Iraqi patients. Mil Med.

[9] Elliott B (2015). Military nurses’ experiences returning from war. J Adv Nurs.

[10] Salmond SW, Echevarri M (2017). Healthcare transformation and changing roles for nursing. Orthop Nurs.

[11] Fukada M (2018). Nursing competency: definition, structure and development. Yonago Acta Med.

[12] AACN. The essentials of baccalaureate education for professional nursing practice. Washington, DC: AACN. 2008. Available from http://www.aacn.nche.edu/education/pdf/BaccEssentials08.pdf.

[13] Reineck C, Finstuen K, Connelly LM, Murdock P (2001). Army nurse readiness instrument: psychometric evaluation and field administration. Mil Med.

[14] Chouhan VS, Srivastava S (2014). Understanding competencies and competency modeling - a literature survey. IOSR-JBM.

[15] Graneheim UH, Lundman B (2004). Qualitative content analysis in nursing research: concepts, procedures and measures to achieve trustworthiness. Nurse Educ Today.

[16] Mohajan HK (2018). Qualitative research methodology in social sciences and related subjects. Journal of Economic Development, Environment and People.

[17] Tong A, Sainsbury P, Craig J (2007). Consolidated criteria for reporting qualitative research (COREQ): A 32-item checklist for interviews and focus groups. Int J Qual Health Care.

[18] Polit D, Beck CT (2012). Nursing research: generating and assessing evidence for nursing practice.

[19] Patrician PA, Shang J, Lake ET (2010). Organizational determinants of work outcomes and quality care ratings among Army Medical Department registered nurses. Res Nurs Health.

[20] Liu Y, Aungsuroch Y (2018). Current literature review of registered nurses’ competency in the global community. J Nurs Scholarship.

[21] Ten Hoeve Y, Jansen G, Roodbol P (2014). The nursing profession: public image, self-concept and professional identity. A discussion paper. J Adv Nurs..

[22] Supamanee T, Krairiksh M, Singhakhumfu L, Turale S (2011). Preliminary clinical nursing leadership competency model: a qualitative study from Thailand. Nurs Health Sci.

[23] Aydin Er R, Sehiralti M, Akpinar A (2017). Attributes of a good nurse. Nurs Ethics.

[24] Poorchangizi B, Borhani F, Abbaszadeh A, Mirzaee M, Farokhzadian J (2019). The importance of professional values from nursing students’ perspective. BMC Nurs.

[25] Weis D, Schank MJ (2009). Development and psychometric evaluation of the Nurses Professional Values Scale–Revised. J Nurs Meas.

[26] Godfrey R, Lilley S, Brewis J (2012). Biceps, bitches and borgs: Reading Jarhead’s representation of the construction of the (masculine) military body. Organ Stud.

[27] Bergman BP, Burdett H, Greenberg N (2014). Service life and beyond-institution or culture?. RUSI Journal.

[28] Ma H, Chihava TN, Fu J, Zhang S, Lei L, Tan J, Lin L, Luo Y (2020). Competencies of military nurse managers: A scoping review and unifying framework. J Nurs Manage.

[29] Quinn B (2017). Role of nursing leadership in providing compassionate care. Nurs Stan.

[30] Wilmoth MC, Shapiro SE (2014). The intentional development of nurses as leaders: a proposed framework. J Nurs Adm.

[31] Butler FK, Hagmann J, Butler EG (1996). Tactical combat casualty care in special operations. Mil Med.

[32] Kreiss Y, Merin O, Peleg K, Levy G, Vinker S, Sagi R, Abargel A, Bartal C, Lin G, Bar A, Bar-On E, Schwaber MJ, Ash N (2010). Early disaster response in Haiti: the Israeli field hospital experience. Ann Intern Med.

[33] Meyer EG, Hall-Clark BN, Hamaoka D, Peterson AL (2015). Assessment of military cultural competence: A pilot study. Acad Psychiatry.

